# Changes with time in skin temperature of the shoulders in healthy controls and a patient with shoulder-hand syndrome

**DOI:** 10.3109/03009734.2010.503354

**Published:** 2010-10-27

**Authors:** Yoichi Koike, Hirotaka Sano, Itaru Imamura, Masako Goto, Masamizu Ooyama, Atushi Kita

**Affiliations:** ^1^Department of Orthopaedic Surgery, Japanese Red Cross Sendai Hospital, Yagiyama Honcyo, Taihaku-Ku, Sendai, MiyagiJapan; ^2^Department of Orthopaedic Surgery, Tohoku University School of Medicine, 1-1, Seiryo, Aoba-Ku, Sendai, MiyagiJapan

**Keywords:** Complications, reflex sympathetic dystrophy (RSD), rotator cuff surgery, shoulder-hand syndrome (SHS), skin temperature

## Abstract

**Background:**

Abnormal skin temperature in the shoulder is caused by various diseases. A thermography is unable to capture temperature changes over time. In contrast, a Thermocron is an effective measuring device to monitor temperature changes over time.

**Purposes:**

The purposes of this study employing a Thermocron were to measure shoulder skin temperature over time in healthy subjects and to detect shoulder skin temperature abnormalities in a patient with shoulder-hand syndrome.

**Subjects and methods:**

Subjects comprised 10 healthy volunteers (20 shoulders; 4 men and 6 women, mean age 54 years). For measurements, a Thermocron was attached on both shoulders. Measurements were made from 21.00 to 07.00 the following morning at 15-minute intervals.

**Results:**

Gradual difference in right and left shoulder skin temperature was observed with the timing of measurements but no significant difference was apparent, i.e. dominant side 34.9 ± 0.8°C, non-dominant side 34.9 ± 0.9°C (*P* = 0.28).

**Presentation of a case with shoulder-hand syndrome:**

A 54-year-old woman with the diagnosis of rotator cuff tear underwent surgical treatment of rotator cuff repair, but the pain of the operated shoulder persisted due to phase 1 shoulder-hand syndrome. In postoperative week 3, skin temperature measurement using Thermocrons demonstrated a significant decrease in temperature on the operated side (affected side 34.3 ± 0.4°C, healthy side 35.2 ± 0.3°C; *P* < 0.05).

**Conclusion:**

The changing of the skin temperature during night-time was successfully recorded both in the healthy subjects and a case with shoulder-hand syndrome using a Thermocron.

## Introduction

Various shoulder diseases cause abnormal skin temperature in the shoulder. Skin temperature of the shoulder is reportedly decreased in rotator cuff tendonitis, frozen shoulder, and reflex sympathetic dystrophy (RSD) (1–3). In contrast, skin temperature is reported to be normal in shoulders with rotator cuff tears (3).

**Figure 1.**
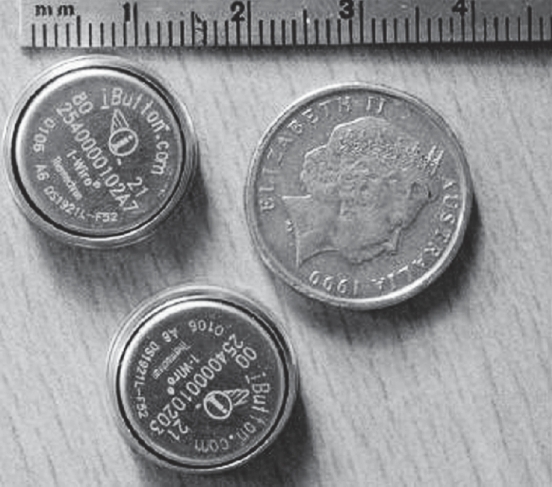
The Thermocron (Maxim Integrated Products, Inc., Sunnyvale, CA, USA) is 15 mm in diameter and is equipped with a temperature sensor, memory, and battery.

To our knowledge, thermography has been widely used for the diagnosis of abnormal skin temperature (1,2,4,5). However, thermography measurements are limited to a short time and so have the disadvantage of being unable to capture temperature changes over time. In contrast, the Thermocron (Maxim Integrated Products, Sunnyvale, CA, USA) is an effective measurement device for monitoring temperature changes over time (6) (Figure 1).

The purposes of this study employing the Thermocron were: 1) to measure shoulder skin temperature over time in a healthy group; and 2) to detect shoulder skin temperature abnormalities in a patient with shoulder-hand syndrome (SHS) (a subtype of RSD) (4,7).

## Subjects and methods

As a healthy group, subjects comprised 10 volunteers (20 shoulders) with no shoulder complaints, including 4 men and 6 women. Mean age was 54 years, and all were right-handed. Medical histories included leg fracture in five, foot fracture in three, and hallux valgus in two. Measurements were made in all subjects during hospitalization for removal of metal implants from the leg. A case with shoulder-hand syndrome was also evaluated and reported as a case presentation.

For measurements, the Thermocron was attached 5 cm below the anterolateral edge of both acromions. Insulating tape (Nitoms Inc., Tokyo, Japan) was applied to the surface of the Thermocron, and an adhesive sheet (PERME-ROLL, Nitto Medical Co. Osaka, Japan) was placed over that surface to avoid peeling off from the skin.

Serial measurements were made from 21.00 to 07.00 the following morning, during what was assumed to be a time at rest. Measurements were made at 15-minute intervals. Another Thermocron was attached to a room wall to monitor temperature and humidity. After measurements, all Thermocrons were removed and the data were collected.

### Statistics

A paired *t* test was used in statistical analysis, and a comparison was made between the dominant and non-dominant shoulders. PASW Statistics v. 18 software (SPSS, Chicago, IL, USA) was used, with a significance level of *P* = 0.05.

### Ethics

The protocol of this study was approved by the ethics board of Japanese Red Cross Sendai Hospital (Registration number R1000245, approved on 20 June 2009) and was conducted in accordance with the Declaration of Helsinki. All patients gave informed consent to participate in this study.

## Results

**Figure 2.**
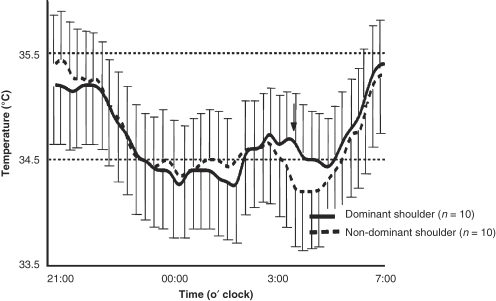
Temperature changes in both shoulders in the healthy group. A gradual difference in dominant (solid line) and non-dominant (broken line) shoulder skin temperature was observed with the timing of measurements, reaching a maximum of 0.6°C (arrow). However, rhythms of the decline and rises in skin temperature were similar in both shoulders. Bar: standard deviation.

Temperature changes in both shoulders in the healthy group are shown in Figure 2. Skin temperature of both shoulders was observed to drop from about 22.00. A temporary rise in skin temperature occurred from about 02.00 to 04.00. The skin temperature of both shoulders remained low until about 05.00 and subsequently increased from about 05.00 to 07.00 (Figure 2).

A gradual difference in right and left shoulder skin temperature was observed, reaching a maximum of 0.6°C (Figure 2, arrow). However, rhythms of the decline and rises in skin temperature were similar in both shoulders, and no significant difference was apparent: dominant side 34.9 ± 0.8°C, non-dominant side 34.9 ± 0.9°C (mean ± standard deviation; *P* = 0.28).

During the experimental period, the temperature and humidity of the room remained constant, indicating a stable environment (temperature: mean 26.2°C, maximum 26.4°C, minimum 26.1°C; humidity: mean 58%, maximum 59%, minimum 57%).

## Case presentation

The patient was a 54-year-old woman who had fallen 10 years earlier on an icy road and injured her right shoulder. At that time, she was diagnosed with a sprain of the acromioclavicular joint and underwent conservative treatment, after which she had no shoulder complaints. Then, without any inciting causes, she had begun to feel pain on the right shoulder starting 6 months before the current presentation.

Physical examinations identified limited range of motion in abduction, external rotation, and extension of the right shoulder. Decreased muscle strength in abduction and external rotation was also identified only in the right shoulder. The shoulder was positive for impingement sign (8) and in the block test (9).

On the visual analogue pain scale (0–10 numeric pain intensity scale) (10), day-time pain was 4 and night-time pain was 9. The score on the Japanese Orthopaedic Association scale (JOA score, maximum 100 points) (11,12) was 48.5 (pain 5, function 9.5, range of motion (ROM) 14, X-ray 5, stability 15). The score on the Disabilities of the Arm, Shoulder and Hand scale (DASH score, maximum 100 points) (13) was 75.

**Figure 3.**
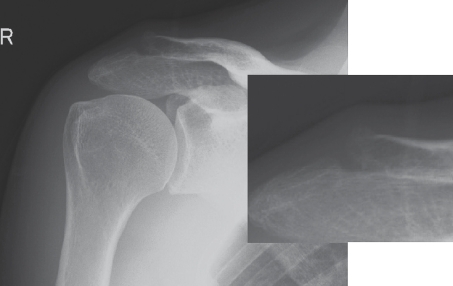
Case presentation. Plain radiographs showing osteosclerosis of the distal end of the clavicle and a cyst in the subchondral bone indicate degenerative arthritis of the acromioclavicular joint.

**Figure 4.**
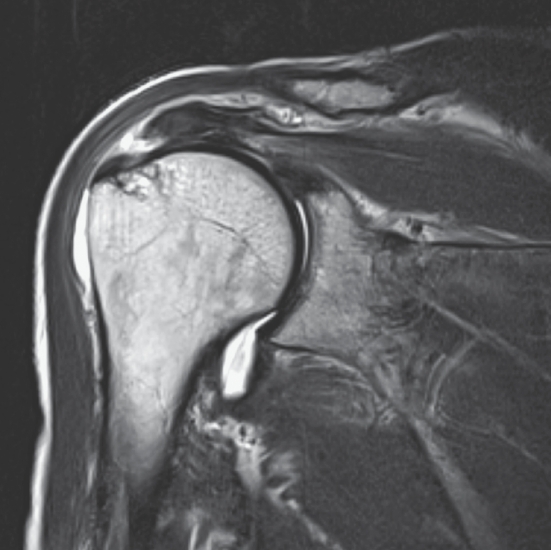
Case presentation. Magnetic resonance image (T2WI) of a shoulder showing rotator cuff tear and glenohumeral effusion.

Radiologically, osteosclerosis of the distal end of the clavicle and a cyst in the subchondral bone were seen on plain radiographs (Figure 3). Magnetic resonance imaging (MRI) demonstrated a presence of rotator cuff tear in the supraspinatus tendon with the collection of joint effusion (Figure 4).

The clinical diagnosis was rotator cuff tear concomitant with degenerative arthritis of the acromioclavicular joint. Surgical treatment was performed, including arthroscopic rotator cuff repair and resection of the distal end of the clavicle.

Unfortunately, early postoperative course was poor in this case; pain during passive movement of the right shoulder and at night-time persisted. Subacromial injection of 50 mg lidocaine (AstraZeneca plc, London, UK) did not work well for pain relief. Intravenous injection of 15 mg of pentazocine (Astellas Pharma Inc., Tokyo, Japan) caused pain to subside only for 1–2 days.

**Figure 5.**
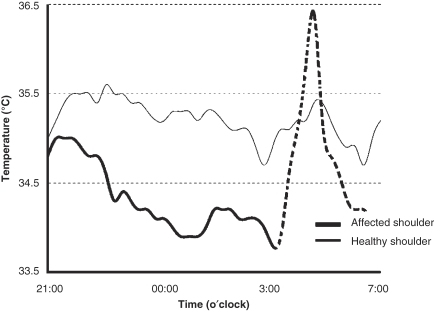
Temperature changes in both shoulders in a patient with shoulder-hand syndrome. In postoperative week 3, a striking decline in temperature on the operated side was seen from about 22.00, lasting until 03.00 the following morning. The patient was awakened by pain at about 03.00. Temperature was elevated by placing a hot pack on the affected shoulder (broken line).

In postoperative week 3, skin temperature of the shoulder was measured using a Thermocron. A striking decline in temperature on the operated side was seen from about 22.00, lasting until 03.00 the following morning (Figure 5). Skin temperature on the affected side decreased significantly in comparison with the healthy side (affected side 34.3 ± 0.4°C, healthy side 35.2 ± 0.3°C; *P* < 0.05). The patient was awakened by pain at about 03.00. Temperature was elevated by placing a hot pack (As-one Corporation, Osaka, Japan) on the right shoulder (Figure 5, broken line).

**Figure 6.**
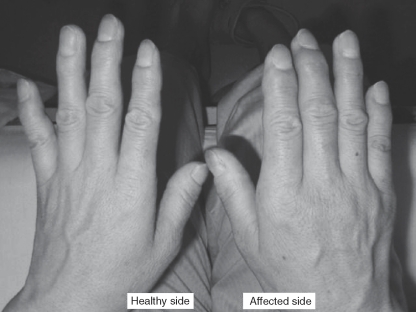
Case presentation. Swelling occurred in the hand on the affected side (right hand) in postoperative week 4, a characteristic and a definitive finding for the diagnosis of phase 1 shoulder-hand syndrome.

Under the running diagnosis of postoperative RSD, oral vitamin C (ascorbic acid, 1500 mg per day) (14) and neurotropin (an extract from cutaneous tissue of rabbit inoculated with vaccinia virus, 4 units per day) (15,16) were prescribed. Additionally, 3.3 mg of dexamethasone (Merck & Co. Inc., Whitehouse Station, NJ, USA) and 50 mg of lidocaine were administered for suprascapular nerve block (17). Swelling (Figure 6) and pain occurred in the hand on the affected side in postoperative week 4, and finally the patient was diagnosed with phase 1 SHS (18).

A gradual decrease in pain was achieved from about postoperative week 6. JOA score in postoperative week 12 was 64 and continued to improve to 81 in week 24. No findings of re-tear in the repaired rotator cuff were identified on MRI in week 24.

## Discussion

Body temperature in the healthy population is known to show circadian fluctuations. The characteristics of these circadian fluctuations are a decrease in body temperature after going to sleep and an elevation after awakening (19,20). The decrease in skin temperature of the shoulder observed at night in the healthy group in this study is also thought to have been affected by circadian fluctuations. Other factors that affect the maintenance of body temperature are referred to as ‘the masking factors’, which include the heat generated by muscle contraction (21,22). Activity in the arms from turning over during the night produces contraction of the shoulder muscle group, which may cause an elevation in the skin temperature of the shoulder.

In the assessment of the temperature on the surface of the shoulder joint, Vecchio et al. defined a right-left difference of <0.5°C as a normal range (2). However, they did not discuss reasons for the right-left difference. The present results suggest that the circadian fluctuation is one factor producing a right-left difference.

To determine the abnormal temperature differences between right and left shoulders, the possibility of false-positive bias (18) must be excluded with the use of statistical techniques. To decrease the risk of bias, it is better to collect data on multiple occasions from measurements made over time. In fact, a comparison of mean values of the skin temperature for both shoulders during the measurement period showed no significant differences in the healthy group.

SHS is reported as painful swelling of the hand that occurs following stroke, myocardial infarction, or trauma (7), and is currently classified as a subtype of RSD. The characteristics of RSD are abnormalities of autonomic nervous regulation in the affected limb (4,5). Abnormalities in autonomic nervous regulation are probably accompanied by abnormal skin temperature. We consider the decreased skin temperature of the shoulder in postoperative week 3 to be the first finding indicating the onset of SHS. The swelling of the hand that occurred in postoperative week 4 was a further characteristic and a definitive finding for the diagnosis of SHS.

This study has several limitations. First, there is a concern about the possibility that skin temperature of the shoulder was affected by undiagnosed neurovascular lesions or bone, joint, or muscle disease. Second, such factors as age, sex, and body fat percentage were not investigated for their effect on skin temperature of the shoulder. Third, only 1 patient with SHS was investigated in the current study. Data should be collected from a larger number of SHS patients following rotator cuff repair to clarify the true diagnostic roles of this method.

In summary, measurements of skin temperature of the shoulders using a Thermocron were reported. Right-left differences in skin temperature of the shoulders were quantified for the healthy group, and the temperature decrease that occurred in a patient with SHS was demonstrated with this technique.
